# Challenging ICU dogmas: a new perspective on venous congestion and preload dependency

**DOI:** 10.1186/s13054-024-04897-0

**Published:** 2024-05-17

**Authors:** Pierre-Grégoire Guinot, Dan Longrois

**Affiliations:** 1 Department of Anaesthesiology and Critical Care Medicine, Dijon University Medical Centre, 21000 Dijon, France; 2 University of Burgundy, LNC UMR1231, 21000 Dijon, France; 3 Anesthesiology and Intensive Care Department, Bichat Claude-Bernard Hospital, Assistance Publique-Hopitaux de Paris - Nord, University of Paris, INSERM U1148, Paris, France

Dear Editor,

We are writing to extend our sincere congratulations to Muñoz et al. for their insightful recent study [1]. Their exploration of the coexistence of preload dependency and venous congestion in ICU patients, as estimated by a specific score, introduces a novel approach to the often-misunderstood dynamics of congestion in critically ill patients. This is particularly important considering the current lack of consensus on definitions of congestion and tools for its estimation. Significantly, their findings suggest that an elevated venous congestion score does not directly correlate with preload dependency, marking a substantial advancement in our understanding of these concepts.

Their article prompts a reevaluation of frequently cited yet commonly misunderstood aspects in ICU settings: the analysis of venous return based on Guyton's model and the application of the VExUS score to estimate venous congestion. These aspects are crucial, especially when considering factors like the etiological context and a comprehensive evaluation of cardiac function, which are often overlooked. The prevailing literature tends to conflate blood volume (stressed vs unstressed), preload dependency, and venous return, leading to the development of flawed concepts such as fluid tolerance, which inaccurately merge the venous return curve with the VExUS score. Arthur C. Guyton's studies on venous return curves, which involved altering right atrial pressure (Pra) through an artificial shunt, provided significant insights into physiology but did not fully encompass the complexities of cardiovascular function, particularly how venous return is intricately linked to cardiac function [2]. Brengelmann criticized Guyton's experiments, asserting that they did not adequately represent venous return dynamics. Instead, he argued that they merely illustrated a functional relationship between blood flow and Pra under specific conditions [3]. He emphasized the significance of considering cardiac dynamics in the study of venous return and Pra. These dynamics reflect reality in human cardiovascular dynamics, with right ventricular contraction contributing up to 70% of venous return [4]. Thus, it is imperative that any interpretation of venous congestion includes considerations of cardiac (dys)function [5]. Regarding the VExUS score, while it can estimate venous congestion, it requires careful interpretation. The score’s value lies in its ability to amalgamate various aspects of organ venous flows, offering a comprehensive view of venous return. Studies indicate that a high score often correlates with cardiac dysfunction in cardiology/cardiac surgery and is linked to acute kidney injury [6]. However, evaluating these parameters without considering their relevance to cardiac function limits the score's utility in clinical contexts beyond cardiology/cardiac surgery.

In an effort to understand how the coupling of venous return and cardiac function, as well as the VExUS score, applies to a broader ICU population, our group conducted an unsupervised statistical analysis [7]. We have demonstrated that clinical parameters commonly used interchangeably to define blood volume, preload dependency, and congestion can be categorized into distinct dimensions. The first dimension distinguishes between 'volume congestion' and 'pressure congestion' parameters, while the second dimension distinguishes between preload dependency states and hemodynamic congestion (as exemplified by NT-pro-BNP and portal pulsatility flow). These findings highlight the fact that parameters typically used interchangeably may not provide consistent information; thus, they should not be used interchangeably. Subsequently, we identified several congestion endotypes (systemic, hemodynamic, and volume overload), each characterized by the etiology of ICU admission. Our findings, in conjunction with those of Muñoz et al., demonstrate a high incidence of acute kidney injury in patients with significant preload dependency and congestion [7]. Abdominal venous congestion, as assessed by the VExUS score, was predominantly observed in systemic congestion cases, including both hemodynamic and volume overload. This indicates that evaluating this score without considering the etiological context and cardiac function is less informative in the complex ICU environment.

The article by Muñoz et al. has some conceptual issues that underscore the importance of incorporating clinical context/etiology into the analysis. Fluid responsiveness indices, such as PPV or SVV, are not always straightforward. For instance, a patient can exhibit positive PPV due to high intra-abdominal pressures, leading to an elevated VExUS score. This highlights the need to consider not only fluid responsiveness indicators but also the clinical context and specific patient endotypes. Therefore, it is critical to emphasize that these aspects—clinical context and endotypes—are fundamental for a comprehensive understanding and management of ICU patients. Our results fully support the observations made by Muñoz et al. and underscore the necessity for a more accurate description and assessment of these endotypes of congestion in future research. This innovative approach could significantly alter our understanding and management of blood volume, preload dependency, and congestion in the ICU, enabling more etiology-specific and personalized therapeutic strategies.

We reiterate our congratulations to the research team for their pioneering work and strongly encourage the scientific community to pursue these vital research endeavors.

## Data Availability

No datasets were generated or analyzed during the current study.
